# A new score for predicting intracranial hemorrhage in patients using anticoagulant drugs

**DOI:** 10.3389/fneur.2025.1475956

**Published:** 2025-01-22

**Authors:** Fuxin Ma, Zhiwei Zeng, Jiana Chen, Chengfu Guan, Wenlin Xu, Chunhua Wang, Jinhua Zhang

**Affiliations:** ^1^Department of Pharmacy, Fujian Maternity and Child Health Hospital, College of Clinical Medicine for Obstetrics & Gynecology and Pediatrics, Fujian Medical University, Fuzhou, China; ^2^Department of Neurosurgery, Fujian Medical University Union Hospital, Fuzhou, China

**Keywords:** intracranial hemorrhage, anticoagulant, prediction, score, risk factors

## Abstract

**Objectives:**

The use of anticoagulants in patients increases the risk of intracranial hemorrhage (ICH). Our aim was to identify factors associated with cerebral hemorrhage in patients using anticoagulants and to develop a predictive model that would provide an effective tool for the clinical assessment of cerebral hemorrhage.

**Methods:**

In our study, indications for patients receiving anticoagulation included AF, VTE, stroke/TIA, arteriosclerosis, peripheral vascular diseases (PVD), prosthetic mechanical valve replacement, etc. Data were obtained from the patient record hospitalization system. Logistic regression, area under the curve (AUC), and bar graphs were used to build predictive models in the development cohort. The models were internally validated, analytically characterized, and calibrated using AUC, calibration curves, and the Hosmer-Lemeshow test.

**Results:**

This single-center retrospective study included 617 patients treated with anticoagulants. Multifactorial analysis showed that male, leukoaraiosis, high risk of falls, APTT ≥ 45.4 s, and FIB ≥ 4.2 g/L were independent risk factors for cerebral hemorrhage, and *β*-blockers were protective factors. The model was constructed using these six factors with an AUC value of 0.883. In the validation cohort, the model had good discriminatory power (AUC = 0.801) and calibration power. Five-fold cross-validation showed Kappa of 0.483.

**Conclusion:**

Predictive models based on a patient’s medical record hospitalization system can be used to identify patients at risk for cerebral hemorrhage. Identifying people at risk can provide proactive interventions for patients.

## 1 Introduction

Anticoagulant drugs, mainly including warfarin and rivaroxaban, are widely used to prevent thromboembolic diseases (such as ischemic stroke) in patients with atrial fibrillation (1, 2). Although anticoagulants have successfully reduced the risk of such events, long-term use of them may be accompanied by an increase in the risk of major bleeding. Intracranial hemorrhage (ICH) is a relatively common type, and the result is catastrophic, often leading to death or severe neurological disability (3, 4).

Intracranial hemorrhage (also known as cerebral hemorrhage) has a huge negative clinical impact because it can increase the risk of death by 50%, and its severity cannot be ignored (5). Compared with the general population, the bleeding rate of patients receiving anticoagulation treatment is significantly higher, and the annual bleeding rate in clinical trials ranges from 1.3 to 7.2% (6). In addition, warfarin-related ICH is 7 to 10 times higher than spontaneous ICH (7). Studies have shown that direct oral anticoagulants (DOACs) are the treatment of choice for atrial fibrillation/venous thromboembolism and have surpassed vitamin K antagonists (VKAs) as the most prescribed anticoagulant worldwide. DOACs have similar or better efficacy/safety and reduced risk of ICH compared to VKAs, but the risk of ICH still cannot be ignored (8). Therefore, an urgent clinical question is how to reliably predict which patients are at high risk of ICH after anticoagulant therapy, i.e., which factors are risk factors for ICH in patients.

Given the widespread use of anticoagulant therapy and the serious consequences of bleeding events, it is important to predict the risk factors for intracranial hemorrhage associated with anticoagulant drugs and to construct a risk score. There are several bleeding risk scores, such as PANWARDS and HAS-BLED scores. PANWARDS score is used to assess the risk of ICH in AF patients with anticoagulant therapy, ignore other patients who may need anticoagulation treatment, such as lower limb vein thrombosis and pulmonary embolism (9). The main outcome of the HAS-BLED score is major bleeding, including gastrointestinal bleeding and ICH˙ It is worth noting that in the latest ESC guidelines for AF, the HAS-BLED score has been removed (10). So the above models used to predict ICH independently may not be accurate (11, 12). Therefore, establishing and using an anticoagulant drug–related ICH model is crucial.

Our study identified risk factors for cerebral hemorrhage associated with anticoagulants and created a score to assess the risk of intracranial hemorrhage. In addition, our score was internally validated to assess the predictive ability of the model. Our aim is to help clinicians better predict the risk of cerebral hemorrhage so that they can optimize anticoagulation therapy and reduce the incidence of intracranial bleeding events.

## 2 Methods

### 2.1 Study design

We retrospectively analyzed patients using anticoagulants from January 2010 to July 2021 in a teaching hospital in southern China. Data were obtained from the patient medical record hospitalization system. The hospital ethics committee approved this study. The study registration number was ChiCTR2000031909, the registration date was April 2020, and the ethical review number was 2020KY087. Because of the retrospective nature of this study, the review committee waived the requirement for patient informed consent. Exclusion criteria: (1) pregnant or lactating women; (2) severe mental illness or mental disorder; (3) intracranial hemorrhage had occurred before anticoagulation therapy, i.e., intracranial hemorrhage was not caused by anticoagulant medication; and (4) insufficient data for subsequent analysis. Patients who received catheter-based anticoagulation therapy were not included in this study.

### 2.2 Data collection

We collect data on each patient from the hospital’s electronic database. The data were clinically organized and recorded by specialized physicians and nurses in a timely manner, including demographic information, lifestyle habits, combination medications, concomitant illnesses, imaging, functional scores, and laboratory markers (13, 14). For clinical use, all continuous variables (e.g., PT, INR, APTT, FIB, TT, d -dimer) were analyzed using the subject’s work characteristics (ROC) curve. Critical values were determined based on the maximum value of the Jordon’s index (Jordon’s index = sensitivity + specificity—1) and transformed into secondary variables.

Relevant clinical indicators were defined as follows: higher risk of falls is defined in detail as the presence of one of the following conditions: age ≥ 80 years; 2 or more falls in the 6 months prior to hospitalization or during this hospitalization; Presence of unsteady gait, joint and/or muscle pain in the lower extremities, visual impairment, etc.; sedative, analgesic, or sleeping medication used within 6 h; alcohol consumption was defined as more than 8u of alcohol per week; Cardiovascular diseases included [atrial fibrillation (AF), coronary heart disease, hypertension, peripheral vascular diseases (PVD)]. Major hemorrhage was defined as bleeding requiring hospitalization, a decrease in hemoglobin level of ≥ 2 g/dL, or the need for transfusion of ≥ 2 units of whole blood or red blood cells (15); Anemia was defined as hemoglobin < 13 g/dL in men or hemoglobin < 12 g/dL in women (16); The definition and evaluation of leukoaraiosis are the same as those in previous studies (13, 17).

### 2.3 Endpoints

The primary outcome of this study was ICH˙ ICH is defined as any primary cranial cavity hemorrhage that is clinically evident (i.e., causes symptoms or signs) and confirmed by brain imaging (CT or MRI brain scan) or autopsy. ICH is categorized according to the site of the hemorrhage as parenchymal or ventricular systemic hemorrhage (cerebral hemorrhage), subarachnoid (arachnoid hemorrhage), subdural (subdural hemorrhage), or extradural (extradural hemorrhage). Relevant definitions are spelled out in previous studies (9, 13).

### 2.4 Statistical analysis

Continuous variables are expressed as mean ± standard deviation (obeying normal distribution) or median (interquartile spacing) (not obeying normal distribution), and categorical variables are expressed as percentages. One-way analyses of categorical variables were performed using the Pearson χ2 test or Fisher exact test, as appropriate. Since a large number of observations may lead to statistical significance of weaker associations, only variables with *p*-values less than 0.2 were further included in the multivariate analysis. Multivariate logistic regression was then used to identify significant factors. The process of model construction comprises three stages: the selection of variables, the construction of the model itself, and the subsequent validation of the model. According to the model’s *β* coefficients, each predictor’s weight was determined. A predictive model for the risk of intracranial hemorrhage was constructed based on the calculated scores for each variable. The calibration plots were evaluated in order to ascertain their calibration power and to perform a Hosmer-Lemeshow test. The calibration plots represent the relationship between the frequency of the results and the predicted probability (18). The area under the receiver operating characteristic (ROC) curve (AUC) is employed for the purpose of quantifying the binary outcomes (hospital admission or non-admission). The ROC curve is constructed by plotting the sensitivity and specificity values for each possible threshold value, thereby creating a continuous representation of the diagnostic accuracy across the entire range of possible cutoffs. The AUC serves to reflect the accuracy of the predictive models in question, and thus allows for comparison between the various models. AUC 0.5 means the model has no discrimination (the proportions of true cases and false positive cases are equal) whereas AUC 1.0 means the model has a perfect discrimination (19). Nomogram was a visual expression of a logistic regression model with a user-friendly interface, better accuracy, and easy-to-understand result, which was one of the most popular research directions (20). We estimated the required sample size based on the event rate and the pre-determined model complexity (the total number of degrees of freedom of the final candidate predictors) as well as the four criteria required for good model performance (21). To avoid overfitting data during the modeling process, the remaining 30% of the data is used for internal validation of the model. All statistical analyses were performed using SPSS software (version 25.0) and R software (version 4.2.0).

## 3 Results

### 3.1 Patient characteristics

**Figure 1:**
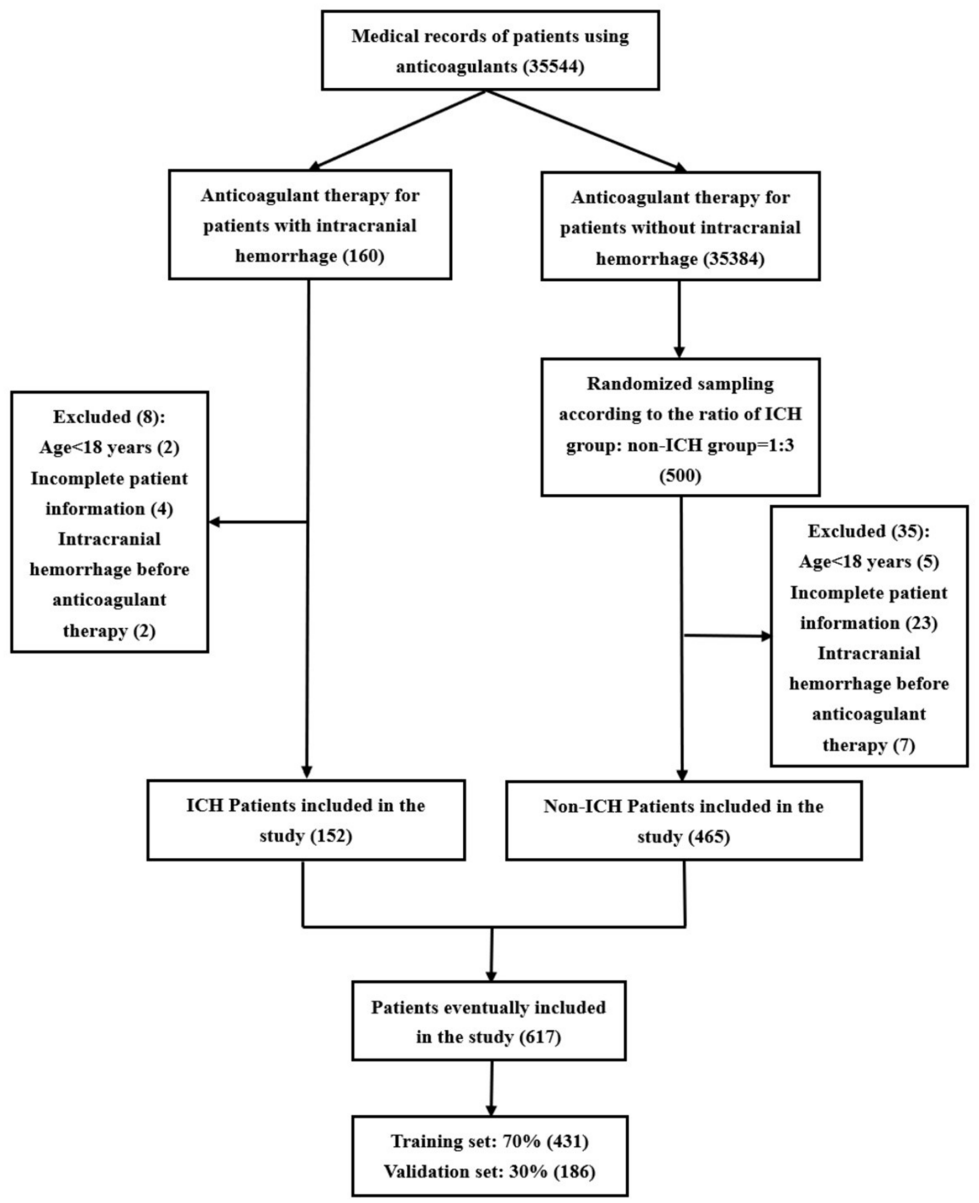
Flowchart of patient screening.

The patient screening flowchart is illustrated in Figure 1. This study included a total of 617 patients, of whom 431 (70%) were used for model construction and 186 (30%) were used for internal validation. A total of 106 cerebral hemorrhages occurred in the development group (*n* = 431), while 46 occurred in the validation group (*n* = 186). This resulted in an incidence rate of 0.43% (152/35,544). Baseline characteristics are presented in Table 1.

**Table 1:** Baseline clinical characteristics of patients.

Demographic and clinical information	*N* = 431
Male, *n* (%)	237 (54.99)
Age, years, median (25–75% quartile)	62 (53–71)
BMI ≥ 28 Kg/m^2^, *n* (%)	37 (8.58)
Smoking, *n* (%)	60 (13.92)
Drinking, *n* (%)	88 (20.42)
Antiplatelet drugs or NSAIDs, *n* (%)	76 (17.63)
Dual antiplatelet therapy, *n* (%)	24 (5.57)
Antihypertensive drugs, *n* (%)	154 (35.73)
Lipid-lowering drugs, *n* (%)	52 (12.06)
Antacid drugs, *n* (%)	22 (5.10)
Antibacterials, *n* (%)	16 (3.71)
β receptor blockers, *n* (%)	63 (14.62)
Hyperlipidemia, *n* (%)	69 (16.01)
Diabetes, *n* (%)	76 (17.63)
Chronic liver disease or pancreatitis, *n* (%)	110 (25.52)
MT, *n* (%)	46 (10.67)
Esophageal varices, *n* (%)	5 (1.16)
History of major bleeding, *n* (%)	27 (6.26)
VTE, *n* (%)	83 (19.26)
Cardiogenic embolism, *n* (%)	22 (5.10)
Small vascular diseases, *n* (%)	115 (26.68)
Lacunar infarction, *n* (%)	78 (18.10)
Cerebral microbleeds, *n* (%)	3 (0.70)
Arteriosclerosis, *n* (%)	78 (18.10)
Cardiovascular diseases, *n* (%)	385 (89.33)
Heart failure or cardiac insufficiency, *n* (%)	249 (57.77)
AF, *n* (%)	210 (48.72)
Coronary heart disease, *n* (%)	82 (19.03)
Hypertension, *n* (%)	175 (40.60)
Valvular heart disease, *n* (%)	229 (53.13)
Pericarditis or infective endocarditis, *n* (%)	95 (22.04)
PVD, *n* (%)	74 (17.17)
Use of artificial heart valves, *n* (%)	143 (33.18)
Cerebrovascular diseases, *n* (%)	117 (27.15)
Stroke or TIA, *n* (%)	112 (25.99)
Intracranial aneurysm, *n* (%)	7 (1.62)
Anemia, *n* (%)	155 (35.96)
Leukoaraiosis, *n* (%)	89 (20.65)
Higher risk of fall, *n* (%)	181 (41.20)
Absence of collateral flow, *n* (%)	6 (1.39)
Middle cerebral artery occlusion, *n* (%)	13 (3.02)
Abnormal NIHSS, *n* (%)	114 (26.45)
Abnormal GCS, *n* (%)	111 (25.75)
Abnormal HAS-BLED, *n* (%)	188 (43.62)
Decreased platelet count, *n* (%)	22 (5.10)
Abnormal liver function, *n* (%)	134 (31.09)
Abnormal renal function, *n* (%)	76 (17.63)
PT, s, median (25–75% quartile)	13.8 (12.8–16.9)
INR, median (25–75% quartile)	1.06 (0.97–1.39)
APTT, s, median (25–75% quartile)	38.4 (35.2–43.7)
FIB, g/L, median (25–75% quartile)	3.69 (3.01–4.50)
TT, s, median (25–75% quartile)	16.9 (16.1–17.9)
D-dimer, ug/mL, median (25–75% quartile)	0.67 (0.30–1.94)

BMI: body mass index; NSAIDs: non-steroidal anti-inflammatory drugs; MT: malignant tumor; VTE: venous thromboembolism; AF: atrial fibrillation; PVD: peripheral vascular diseases; TIA: transient ischemic attack; NIHSS: national institutes of health stroke scale; GCS: glasgow coma scale; PT: prothrombin time; INR: international normalized ratio; APTT: activated partial thromboplastin time; FIB: fibrinogen; TT: thrombin time.

### 3.2 Convert continuous variable to classified variable

The Yoden index was calculated for continuous variables based on the results of the ROC curve analysis. The Yoden index is defined as sensitivity plus specificity minus one. The maximum value of the Yoden index is used as the cutoff value. Once the cutoff value was determined, the continuous variables were converted to secondary variables. The cutoff values for each continuous variable are presented in Table 2.

**Table 2:** The cut-off value/threshold value of each continuous variable.

Variables	Sensitivity	1- specificity	Youden index	Cut-off value
PT	0.774	0.492	0.266	13.45
INR	0.792	0.471	0.263	1.025
APTT	0.377	0.849	0.226	45.35
FIB	0.519	0.729	0.248	4.195
TT	0.264	0.834	0.098	18.25
D-dimer	0.802	0.394	0.196	0.425

PT: prothrombin time; INR: international normalized ratio; APTT: activated partial thromboplastin time; FIB: fibrinogen; TT: thrombin time.

### 3.3 Development of the risk score

In total, 53 variables were available for analysis and Supplementary Table S1 presents the 36 most significant variables from the univariable analyses. In univariate analysis, males (*p* = 0.050), age ≥ 75 years old (*p* = 0.001), dual antiplatelet therapy (*p* = 0.046), *β* receptor blockers (*p* = 0.003), diabetes (*p* = 0.002), chronic liver disease or pancreatitis (*p* = 0.075), esophageal varices (*p* = 0.199), history of major bleeding (*p* = 0.003), VTE (*p* = 0.062), cardiogenic embolism (*p* = 0.083), small vascular diseases (*p* < 0.001), lacunar infarction (*p* < 0.001), cerebral microbleeds (*p* = 0.089), arteriosclerosis (*p* < 0.001), cardiovascular diseases (*p* = 0.001), AF (*p* = 0.036), hypertension (*p* < 0.001), PVD (p < 0.001), cerebrovascular diseases (*p* < 0.001), stroke or TIA (*p* < 0.001), intracranial aneurysm (*p* < 0.001), leukoaraiosis (*p* < 0.001), higher risk of fall (*p* < 0.001), absence of collateral flow (*p* < 0.001), middle cerebral artery occlusion (*p* < 0.001), abnormal liver function (*p* = 0.029), abnormal renal function (*p* = 0.032), abnormal NIHSS (*p* < 0.001), abnormal GCS (*p* = 0.049), abnormal HAS-BLED (*p* < 0.001), PT ≥ 13.5 s (*p* < 0.001), INR ≥ 1.03 (*p* < 0.001), APTT ≥ 45.4 s (*p* < 0.001), FIB ≥ 4.20 g/L (*p* < 0.001), TT ≥ 18.3 s (*p* = 0.026) and D-dimer ≥ 0.43 ug/mL (*p* < 0.001), 36 factors with *p* < 0.2, statistically significant and included in multifactorial analysis.

The results from the multivariable final predictive model are presented in Supplementary Table S2. In the multivariate analysis, six factors were included in the final model (Table 3). Male (OR, 1.927; 95% CI, 1.064–3.490; *p* = 0.030), leukoaraiosis (OR, 10.039; 95% CI, 5.184–19.439; *p* < 0.001), higher risk of fall (OR, 5.963; 95% CI, 3.249–10.943; *p* < 0.001), APTT ≥ 45.4 s (OR, 3.022; 95% CI, 1.582–5.772; *p* = 0.001) and FIB ≥ 4.2 g/L (OR, 2.137; 95% CI, 1.190–3.841; *p* = 0.011) were associated with an increased risk of ICH˙ *β* receptor blockers were associated with a reduced risk of ICH (OR, 0.145; 95% CI, 0.048–0.439; *p* = 0.001).

**Table 3:** Risk scoring scale for anticoagulant drugs-related ICH.

Variables	β	OR (95% CI)	*p* value
Male	0.656	1.927 (1.064–3.490)	0.030
β receptor blockers	−1.931	0.145 (0.048–0.439)	0.001
Leukoaraiosis	2.306	10.039 (5.184–19.439)	<0.001
Higher risk of fall	1.786	5.963 (3.249–10.943)	<0.001
APTT ≥ 45.4 s	1.106	3.022 (1.582–5.772)	0.001
FIB ≥ 4.2 g/L	0.760	2.137 (1.190–3.841)	0.011

APTT: activated partial thromboplastin time; FIB: fibrinogen.

**Figure 2:**
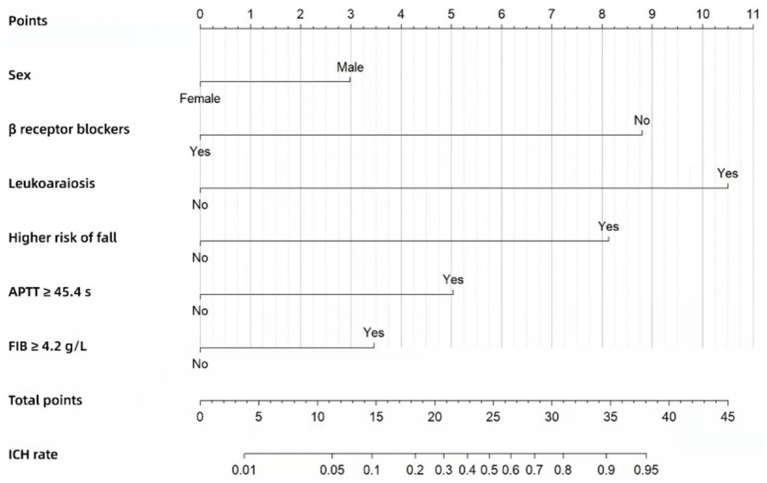
Nomogram for predicting the risk of ICH in the development cohort.

The Nomogram (Figure 2) was also constructed based on the beta regression coefficients in multivariate analysis and scored for the six factors, with a total score at least 28.5 considered as high ICH risk. The ICH risk score—which we named the Alfalfa-Anticoagulant-ICH score (“Alfalfa” is the name of our team, representing happiness and luck). It can be seen from the Nomogram that 3 points for males, 9 points for not using *β* receptor blockers (0 points for use), 10.5 points for leukoaraiosis, 8 points for higher risk of fall, 5 points for APTT ≥ 45.4 s, and 3.5 points for FIB ≥ 4.2 g/L. The total score of the model is 0–39. When the score is ≥ 14.5, 21, 24.5, 28.5 and 34.5, the probability of ICH is 10, 30, 50, 70 and 90%, respectively. We define score ≥ 28.5 (probability of ICH ≥ 70%) as a high-risk group, 21 ≤ score < 28.5 (30% ≤ probability of ICH < 70%) as a moderate-risk group, and score < 21 (probability of ICH < 30%) as a low-risk group.

### 3.4 Model validation

**Figure 3:**
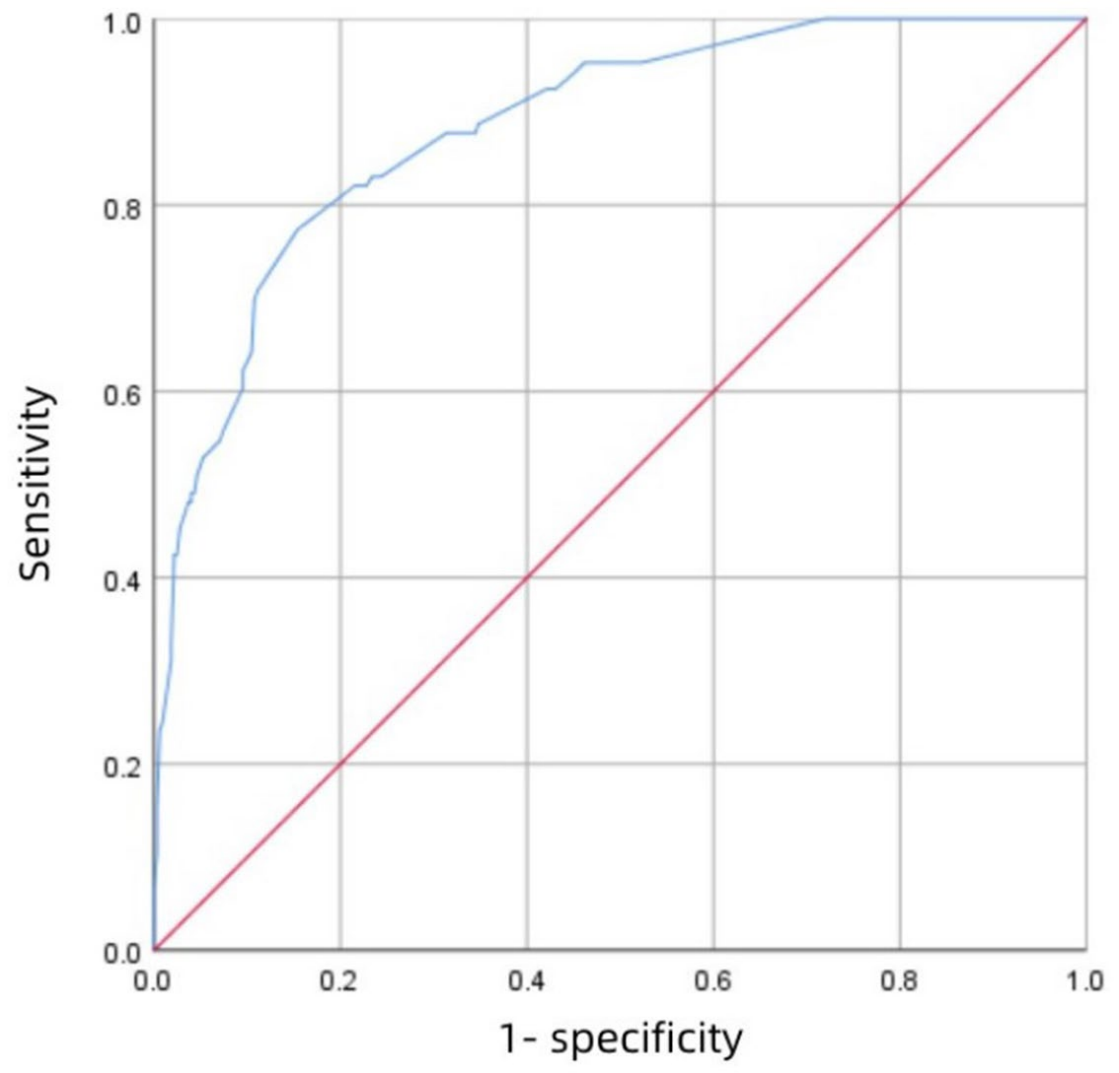
The ROC curve of the development cohort.

**Figure 4:**
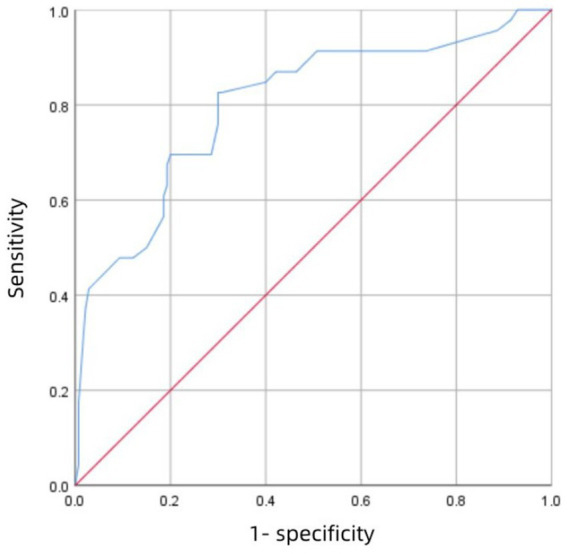
The ROC curve of the validation cohort.

Figure 3 shows the receiver operating characteristic (ROC) curve of score with an AUC value of 0.883 (95% CI 0.847–0.918; P<0.001), which has a strong predictive power. Figure 4 shows the ROC curve of the internal validation cohort with an AUC value of 0.801 (95%CI 0.723–0.879; *p* < 0.001). We performed a five-fold cross-validation on the 617 individuals included in the study, which showed a precision of 0.83, a Kappa of 0.483, an AUC of 0.87, and a good generalization ability. We performed sensitivity analyses for variables such as dual antiplatelet therapy and hepatic insufficiency to assess whether model parameter estimates were accurate. We used logistic regression to correct for confounders. Three separate logistic regression models were developed; the first model did not correct for confounders and had an AUC value of 0.883; the second model corrected for dual antiplatelet therapy had an AUC value of 0.983; the third model corrected for hepatic insufficiency on top of the second model with an AUC value of 0.978, which shows a change in the predictive performance of the model after correction for confounders.

**Figure 5:**
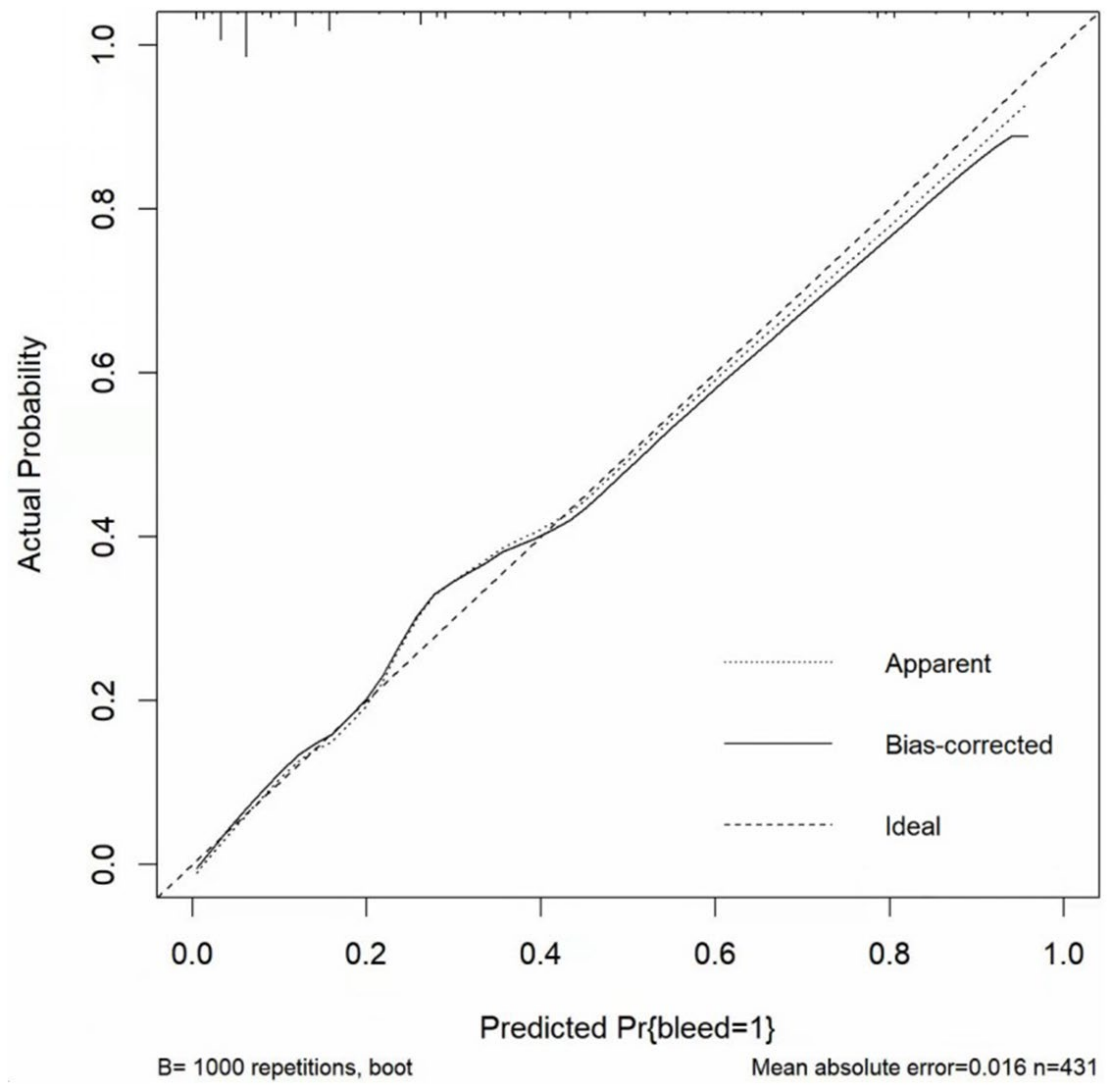
Calibration curve.

**Figure 6:**
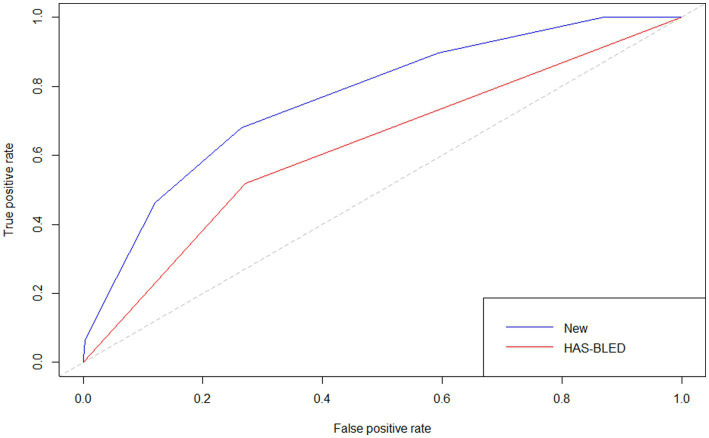
The receiver operating characteristic curves for validation of two scores.

The *p* value for the Hosmer–Lemeshow test was 0.905. The calibration curve (Figure 5) showed the model may have good calibration capability. We compared the Alfalfa-Anticoagulant-ICH score to the classic HAS-BLED score. The results showed that AUC_New score_> AUC_HAS-BLED_ (Figure 6). This demonstrates the better performance of the new score.

## 4 Discussion

### 4.1 Summary of findings

We used administrative routine healthcare data in order to develop a prediction model for patients. Male, leukoaraiosis, higher risk of fall, APTT ≥ 45.4 s, FIB ≥ 4.2 g/L and *β* receptor blockers were the most important variables for the model. Therefore, in addition to anticoagulant drugs that can affect ICH, other risk factors such as brain white matter damage should also be paid attention to during treatment. Regularly detect the four coagulation items and prevent patients from falling to reduce the occurrence of ICH.

### 4.2 Implications for practice

At present, many models have been widely used in daily practice, such as PANWARDS, HAS-BLED and QBleed scores, etc. (9, 11–13, 22). However, our findings indicate that some of the factors identified as potentially associated with cerebral hemorrhage were not included or taken into account in the development of most models. These factors include males, a higher risk of falls, APTT, and beta-blockers.

In this study, we found that male is a risk factor for intracranial hemorrhage. However, there is no prediction model for anticoagulant-related ICH to include this risk factor. This is an interesting phenomenon, which may be of great significance for anticoagulant-related intracranial hemorrhage. Compared with the female, males have more bad living habits (such as heavy smoking, drinking, etc.), and heavy family burden leads to overwork and staying up late, as well as emotional excitement, blood pressure is not easy to control, which are important incentives for the male to have intracranial hemorrhage. In fact, studies have confirmed that the bleeding caused by anticoagulant therapy is related in males, but it is targeted at patients using antiplatelet drugs (23–25). Therefore, more research is needed in the future to confirm our view. Male patients should also use anticoagulants with caution, and pay attention to the choice of drugs and dosage adjustment.

Our study also concluded that a higher risk of falls is associated with anticoagulant-related intracranial hemorrhage, which is consistent with the results of Paciaroni et al. (26). In particular, elderly patients have a higher risk of falling, which may make them face a higher ICH risk (27). Therefore, patients should exercise moderately, preferably accompanied by family members, and try to avoid falling. In addition, we also found that leukoaraiosis may be one of the risk factors for intracranial hemorrhage. Some studies have shown that leukoaraiosis (computed tomography-defined white matter hypodensity) gives a special risk of warfarin-related ICH after stroke. In this study, the presence of leukoaraiosis was associated with a significantly increased risk ratio for intracranial hemorrhage. Even after controlling age, blood pressure and INR, this effect is still significant (28). Leukoaraiosis is the most important predictor in this model, but this factor has not been included in the existing models. Clinical workers should pay enough attention to it. Doctors should carefully evaluate the white matter damage of the brain according to the results of imaging (such as CT or MRI) before giving anticoagulant drugs to patients, and fully weigh the advantages and disadvantages to make a decision.

Additionally, our findings indicated a correlation between APTT and fibrinogen levels and the incidence of cerebral hemorrhage. Nevertheless, only a subset of studies incorporated these two variables into the final model. In comparison to other models, our study gave due consideration to the significance of coagulation indices in the context of bleeding disorders, and incorporated them into the analytical framework. Abnormality of coagulation function will increase the risk of ICH and cause various cardiovascular and cerebrovascular diseases, such as myocardial infarction or stroke, which has been confirmed by research (29). Therefore, doctors and nurses should pay attention to the patient’s coagulation indicators, and patients should also be followed up regularly to monitor the four coagulation items.

Unlike other models, our model included *β* receptor blockers as a factor, which is thought to be associated with a reduced risk of ICH˙ Recently, a study based on Scottish adults concluded that: The risk of new ICH or persistent/progressive focal neurological deficit is low in patients with cerebral cavernous vascular malformation treated with *β* receptor blockers (30), which is similar to our results. In addition, other studies have found that early oral β Receptor blockers, whether high dose (defined as an equivalent dose of metoprolol ≥ 50 mg/day) or low dose (equivalent dose of metoprolol < 50 mg/day), can reduce the risk of major bleeding by 52% (22). The antihypertensive effect and the reduction of myocardial oxygen consumption of β receptor blockers significantly reduce the incidence of cardiovascular and cerebrovascular diseases, bringing more benefits to patients. Nevertheless, our conclusions need to be confirmed by further RCT studies and prospective studies.

### 4.3 Strengths and limitations

The main strength of this study in comparison to earlier smaller and more selected studies is rigorous methods and included comprehensive factors. In addition, the incorporation of a nomogram enhances the comprehensiveness and precision of the model. Ultimately, the model demonstrated enhanced discrimination (AUC = 0.883), superior accuracy and calibration (*p*-value of 0.905 for the Hosmer-Lemeshow test). These advantages may have clinical implications for the prevention of anticoagulant-associated cerebral hemorrhage.

The validity of the prediction tool is of paramount importance, as it may prove useful in a broader clinical context, for instance in other countries with comparable administrative health data structures. The lack of data from other countries represents a potential limitation of this study. Secondly, the main anticoagulants included in our study were warfarin, rivaroxaban, dabigatran, and low-molecular heparin. However, the type of anticoagulant may act as a potential influencing factor. In future studies, it would be interesting to analyze the effect of the type of anticoagulant on ICH˙ Furthermore, it is advantageous to construct bleeding scores in relation to various anticoagulant medications. Finally, our scoring tool has not yet been applied to the clinic on a large scale, so its versatility also requires further investigation in large-scale prospective multicenter research studies.

## 5 Conclusion

We present a clinically useful prediction model with acceptable accuracy for patients at risk of ICH. The methodology and models employed are sufficiently generalizable and can be readily implemented in the majority of healthcare systems. The prediction of patients at risk of ICH may represent a valuable approach for the future healthcare system to address the growing demand for care. However, its implementation in clinical practice must be guided by sound clinical judgment. It may prove an effective tool in reducing the incidence of ICH in patients undergoing anticoagulant therapy.

## Data Availability

The original contributions presented in the study are included in the article/Supplementary material, further inquiries can be directed to the corresponding author.

## References

[1] Connolly SJ Laupacis A Gent M Roberts RS Cairns JA Joyner C. Canadian atrial fibrillation anticoagulation (CAFA) study. J Am Coll Cardiol. (1991) 18:349–55. doi: 10.1016/0735-1097(91)90585-W, PMID: 1856403

[2] Ezekowitz MD Bridgers SL James KE Carliner NH Colling CL Gornick CC. Warfarin in the prevention of stroke associated with nonrheumatic atrial fibrillation. Veterans affairs stroke prevention in nonrheumatic atrial fibrillation investigators. N Engl J Med. (1992) 327:1406–12. doi: 10.1056/NEJM199211123272002, PMID: 1406859

[3] Banerjee A Lane DA Torp-Pedersen C Lip GY. Net clinical benefit of new oral anticoagulants (dabigatran, rivaroxaban, apixaban) versus no treatment in a 'real world' atrial fibrillation population: a modelling analysis based on a nationwide cohort study. Thromb Haemost. (2012) 107:584–9. doi: 10.1160/TH11-11-0784, PMID: 22186961

[4] Hylek EM Singer DE. Risk factors for intracranial hemorrhage in outpatients taking warfarin. Ann Intern Med. (1994) 120:897–902. doi: 10.7326/0003-4819-120-11-199406010-00001 8172435

[5] Gage BF Yan Y Milligan PE Waterman AD Culverhouse R Rich MW. Clinical classification schemes for predicting hemorrhage: results from the National Registry of atrial fibrillation (NRAF). Am Heart J. (2006) 151:713–9. doi: 10.1016/j.ahj.2005.04.017, PMID: 16504638

[6] Yung D Kapral MK Asllani E Fang J Lee DS. Investigators of the registry of the Canadian stroke network. Reinitiation of anticoagulation after warfarin-associated intracranial hemorrhage and mortality risk: the best practice for reinitiating anticoagulation therapy after intracranial bleeding (BRAIN) study. Can J Cardiol. (2012) 28:33–9. doi: 10.1016/j.cjca.2011.10.002, PMID: 22153256

[7] Rosand J Eckman MH Knudsen KA Singer DE Greenberg SM. The effect of warfarin and intensity of anticoagulation on outcome of intracerebral hemorrhage. Arch Intern Med. (2004) 164:880–4. doi: 10.1001/archinte.164.8.880, PMID: 15111374

[8] Ballestri S Romagnoli E Arioli D Coluccio V Marrazzo A Athanasiou A. Risk and Management of Bleeding Complications with direct Oral anticoagulants in patients with atrial fibrillation and venous thromboembolism: a narrative review. Adv Ther. (2023) 40:41–66. doi: 10.1007/s12325-022-02333-9, PMID: 36244055 PMC9569921

[9] Hankey GJ Stevens SR Piccini JP Lokhnygina Y Mahaffey KW Halperin JL. Intracranial hemorrhage among patients with atrial fibrillation anticoagulated with warfarin or rivaroxaban: the rivaroxaban once daily, oral, direct factor Xa inhibition compared with vitamin K antagonism for prevention of stroke and embolism trial in atrial fibrillation. Stroke. (2014) 45:1304–12. doi: 10.1161/STROKEAHA.113.004506 24743444

[10] van I Rienstra M Bunting KV Casado-Arroyo R Caso V Crijns H. 2024 ESC guidelines for the management of atrial fibrillation developed in collaboration with the European Association for Cardio-Thoracic Surgery (EACTS). Eur Heart J. (2024) 45:3314–414. doi: 10.1093/eurheartj/ehae176, PMID: 39210723

[11] Pisters R Lane DA Nieuwlaat R de Vos CB Crijns HJ Lip GY. A novel user-friendly score (HAS-BLED) to assess 1-year risk of major bleeding in patients with atrial fibrillation: the euro heart survey. Chest. (2010) 138:1093–100. doi: 10.1378/chest.10-0134, PMID: 20299623

[12] Hippisley-Cox J Coupland C. Predicting risk of upper gastrointestinal bleed and intracranial bleed with anticoagulants: cohort study to derive and validate the QBleed scores. BMJ. (2014) 349:g4606. doi: 10.1136/bmj.g4606, PMID: 25069704 PMC4113281

[13] Ma F Zeng Z Chen J Zhang J. A new score for predicting intracranial hemorrhage in patients using antiplatelet drugs. Ann Hematol. (2024) 103:2511–21. doi: 10.1007/s00277-024-05734-8, PMID: 38630131

[14] Zeng Z Chen J Qian J Ma F Lv M Zhang J. Risk factors for anticoagulant-associated intracranial hemorrhage: a systematic review and Meta-analysis. Neurocrit Care. (2023) 38:812–20. doi: 10.1007/s12028-022-01671-4, PMID: 36670269

[15] Schulman S Kearon C. Subcommittee on control of anticoagulation of the scientific and standardization Committee of the International Society on thrombosis and Haemostasis. Definition of major bleeding in clinical investigations of antihemostatic medicinal products in non-surgical patients. J Thromb Haemost. (2005) 3:692–4. doi: 10.1111/j.1538-7836.2005.01204.x 15842354

[16] Diener HC Bogousslavsky J Brass LM Cimminiello C Csiba L Kaste M. Aspirin and clopidogrel compared with clopidogrel alone after recent ischaemic stroke or transient ischaemic attack in high-risk patients (MATCH): randomised, double-blind, placebo-controlled trial. Lancet. (2004) 364:331–7. doi: 10.1016/S0140-6736(04)16721-4, PMID: 15276392

[17] Smith EE Gurol ME Eng JA Engel CR Nguyen TN Rosand J. White matter lesions, cognition, and reurrent hemorrhage in lobar intracerebral hemorrhage. Neurology. (2004) 63:1606–12. doi: 10.1212/01.WNL.0000142966.22886.20, PMID: 15534243

[18] Steyerberg EW Bleeker SE Moll HA Grobbee DE Moons KG. Internal and external validation of predictive models: a simulation study of bias and precision in small samples. J Clin Epidemiol. (2003) 56:441–7. doi: 10.1016/S0895-4356(03)00047-7, PMID: 12812818

[19] Radadiya D Devani K Brahmbhatt B Reddy C. Major gastrointestinal bleeding risk with direct oral anticoagulants: does type and dose matter?—a systematic review and network meta-analysis. Eur J Gastroenterol Hepatol. (2021) 33:e50–8. doi: 10.1097/MEG.0000000000002035, PMID: 33470705

[20] Sun F Han B Chen Y Gao Y Shen M. Development and external validation of a model for predicting adverse outcomes in women with preeclampsia: a retrospective study from two trans-regional centers in China. Pregnan Hypertens. (2021) 26:133–40. doi: 10.1016/j.preghy.2021.10.008, PMID: 34794010

[21] Riley RD Ensor J Snell KIE Harrell FE Jr Martin GP Reitsma JB. Calculating the sample size required for developing a clinical prediction model. BMJ. (2020) 368:m441. doi: 10.1136/bmj.m441 32188600

[22] Chen J Lv M Xu W Zhang F Huang N Chen X. New score for predicting major bleeding in patients with atrial fibrillation using direct oral anticoagulants. Int J Cardiol. (2023) 376:56–61. doi: 10.1016/j.ijcard.2023.02.017, PMID: 36791968

[23] Hilkens NA Algra A Diener HC Reitsma JB Bath PM Csiba L. Predicting major bleeding in patients with noncardioembolic stroke on antiplatelets: S2TOP-BLEED. Neurology. (2017) 89:936–43. doi: 10.1212/WNL.0000000000004289, PMID: 28768848 PMC5683104

[24] Amarenco P Sissani L Labreuche J Vicaut E Bousser MG Chamorro A. Algra a; PERFORM and PRoFESS committees and investigators. The intracranial-B2LEED3S score and the risk of intracranial hemorrhage in ischemic stroke patients under antiplatelet treatment. Cerebrovasc Dis. (2017) 43:145–51. doi: 10.1159/000453459, PMID: 28088798

[25] Paciaroni M Agnelli G Giustozzi M Caso V Toso E Angelini F. Risk factors for intracerebral hemorrhage in patients with atrial fibrillation on non-vitamin K antagonist Oral anticoagulants for stroke prevention. Stroke. (2021) 52:1450–4. doi: 10.1161/STROKEAHA.120.031827, PMID: 33657853 PMC10561687

[26] Tinetti ME Williams CS. Falls, injuries due to falls, and the risk of admission to a nursing home. N Engl J Med. (1997) 337:1279–84. doi: 10.1056/NEJM199710303371806, PMID: 9345078

[27] Gorter JW. Major bleeding during anticoagulation after cerebral ischemia: patterns and risk factors. Stroke prevention in reversible ischemia trial (SPIRIT). European atrial fibrillation trial (EAFT) study groups. Neurology. (1999) 53:1319–27. doi: 10.1212/WNL.53.6.1319 10522891

[28] Xu DX Du WT Li X Wu ZX Yu GF. D-dimer/fibrinogen ratio for the prediction of progressive hemorrhagic injury after traumatic brain injury. Clin Chim Acta. (2020) 507:143–8. doi: 10.1016/j.cca.2020.04.022, PMID: 32333859

[29] Zuurbier SM Hickman CR Rinkel LA Berg R Sure U al-Shahi Salman R. Association between Beta-blocker or statin drug use and the risk of hemorrhage from cerebral cavernous malformations. Stroke. (2022) 53:2521–7. doi: 10.1161/STROKEAHA.121.037009, PMID: 35410492 PMC9311291

[30] Xu S Li Z Yang T Li L Song X Hao Y. Association between early Oral β-blocker therapy and risk for in-hospital major bleeding after percutaneous coronary intervention for acute coronary syndrome: Findings from CCC-ACS project [published online ahead of print, 2022 Jun 17]. Eur Heart J Qual Care Clin Outcomes. (2022):qcac036. doi: 10.1093/ehjqcco/qcac036, PMID: 35713509

