# Supplementary material for: A new score for predicting intracranial hemorrhage in patients using anticoagulant drugs
Source: Front Neurol. 2025 Jan 22;16:1475956. doi: 10.3389/fneur.2025.1475956 (PMC11794049; doi:10.3389/fneur.2025.1475956)
Supplement: Supplementary file 1 [file Table_1.DOCX]

**Supplementary Table 1. Results of univariate analysis**

| **Variables** | **Category** | **ICH group (n=106)**  **N (%)** | **Non-ICH group**  **(n=325)**  **N (%)** | **χ2** **value** | **P** **value** |
| --- | --- | --- | --- | --- | --- |
| Sex | Male | 67 (63.21) | 170 (52.31) | 3.837 | 0.050 |
|  | Female | 39 (36.79) | 155 (47.69) |  |  |
| Age | ≥75 years old | 31 (29.25) | 48 (14.77) | 11.189 | 0.001 |
|  | <75 years old | 75 (70.75) | 277 (85.23) |  |  |
| BMI | ≥28 kg/m^2^ | 8 (7.55) | 29 (8.92) | 0.193 | 0.661 |
|  | <28 kg/m^2^ | 98 (92.45) | 296 (91.08) |  |  |
| Smoking | Yes | 17 (16.04) | 43 (13.23) | 0.526 | 0.468 |
|  | No | 89 (83.96) | 282 (86.77) |  |  |
| Drinking | Yes | 18 (16.98) | 70 (21.54) | 1.022 | 0.312 |
|  | No | 88 (83.02) | 255 (78.46) |  |  |
| Antiplatelet drugs or NSAIDs | Yes | 22 (20.75) | 54 (16.62) | 0.943 | 0.332 |
|  | No | 84 (79.25) | 271 (83.38) |  |  |
| Dual antiplatelet therapy | Yes | 10 (9.43) | 14 (4.31) | 3.995 | 0.046 |
|  | No | 96 (90.57) | 311 (95.69) |  |  |
| Antacid drugs | Yes | 4 (3.77) | 18 (5.54) | 0.514 | 0.473 |
|  | No | 102 (96.23) | 307 (94.46) |  |  |
| Lipid-lowering drugs | Yes | 10 (9.43) | 42 (12.92) | 0.917 | 0.338 |
|  | No | 96 (90.57) | 283 (87.08) |  |  |
| Beta blockers | Yes | 6 (5.66) | 57 (17.54) | 9.036 | 0.003 |
|  | No | 100 (94.34) | 268 (82.46) |  |  |
| Antibacterials | Yes | 6 (5.66) | 10 (3.08) | 1.492 | 0.222 |
|  | No | 100 (94.34) | 315 (96.92) |  |  |
| Antihypertensive drugs | Yes | 36 (33.96) | 118 (36.31) | 0.191 | 0.662 |
|  | No | 70 (66.04) | 207 (63.69) |  |  |
| Hyperlipidemia | Yes | 20 (18.87) | 49 (15.08) | 0.854 | 0.355 |
|  | No | 86 (81.13) | 276 (84.92) |  |  |
| Diabetes | Yes | 29 (27.36) | 47 (14.46) | 9.154 | 0.002 |
|  | No | 77 (72.64) | 278 (85.54) |  |  |
| Chronic liver disease or pancreatitis | Yes | 34 (32.08) | 76 (23.38) | 3.176 | 0.075 |
|  | No | 72 (67.92) | 249 (76.62) |  |  |
| MT | Yes | 11 (10.38) | 35 (10.77) | 0.013 | 0.910 |
|  | No | 95 (89.62) | 290 (89.23) |  |  |
| Esophageal varices | Yes | 0 (0.00) | 5 (1.54) | 1.650 | 0.199 |
|  | No | 106 (100.00) | 320 (98.46) |  |  |
| History of major bleeding | Yes | 13 (12.26) | 14 (4.31) | 8.617 | 0.003 |
|  | No | 93 (87.74) | 311 (95.69) |  |  |
| VTE | Yes | 27 (25.47) | 56 (17.23) | 3.491 | 0.062 |
|  | No | 79 (74.53) | 269 (82.77) |  |  |
| Cardiogenic embolism | Yes | 2 (1.89) | 20 (6.15) | 3.005 | 0.083 |
|  | No | 104 (98.11) | 305 (93.85) |  |  |
| Small vascular diseases | Yes | 58 (54.72) | 57 (17.54) | 56.476 | ＜0.001 |
|  | No | 48 (45.28) | 268 (82.46) |  |  |
| Lacunar infarction | Yes | 52 (49.06) | 26 (8.00) | 90.900 | ＜0.001 |
|  | No | 54 (50.94) | 299 (92.00) |  |  |
| Cerebral microbleeds | Yes | 2 (1.89) | 1 (0.31) | 2.884 | 0.089 |
|  | No | 104 (98.11) | 324 (99.69) |  |  |
| Arteriosclerosis | Yes | 34 (32.08) | 44 (13.54) | 18.530 | ＜0.001 |
|  | No | 72 (67.92) | 281 (86.46) |  |  |
| Cardiovascular diseases | Yes | 104 (98.11) | 281 (86.46) | 11.382 | 0.001 |
|  | No | 2 (1.89) | 44 (13.54) |  |  |
| Heart failure or cardiac insufficiency | Yes | 57 (53.77) | 192 (59.08) | 0.921 | 0.337 |
|  | No | 49 (46.23) | 133 (40.92) |  |  |
| AF | Yes | 61 (57.55) | 149 (45.85) | 4.380 | 0.036 |
|  | No | 45 (42.45) | 176 (54.15) |  |  |
| Coronary heart disease | Yes | 19 (17.92) | 63 (19.38) | 0.111 | 0.739 |
|  | No | 87 (82.08) | 262 (80.62) |  |  |
| Hypertension | Yes | 64 (60.38) | 111 (34.15) | 22.791 | ＜0.001 |
|  | No | 42 (39.62) | 214 (65.85) |  |  |
| Valvular heart disease | Yes | 53 (50.00) | 176 (54.15) | 0.554 | 0.457 |
|  | No | 53 (50.00) | 149 (45.85) |  |  |
| Pericarditis or infective endocarditis | Yes | 26 (24.53) | 69 (21.23) | 0.506 | 0.477 |
|  | No | 80 (75.46) | 256 (78.77) |  |  |
| PVD | Yes | 30 (28.30) | 44 (13.54) | 12.250 | ＜0.001 |
|  | No | 76 (71.70) | 281 (86.46) |  |  |
| Use of artificial heart valves | Yes | 33 (31.13) | 110 (33.85) | 0.266 | 0.606 |
|  | No | 73 (68.87) | 215 (66.15) |  |  |
| Cerebrovascular diseases | Yes | 70 (66.04) | 47 (14.46) | 107.510 | ＜0.001 |
|  | No | 36 (33.96) | 278 (85.54) |  |  |
| Stroke or TIA | Yes | 67 (63.21) | 45 (13.85) | 101.259 | ＜0.001 |
|  | No | 39 (36.79) | 280 (86.15) |  |  |
| Intracranial aneurysm | Yes | 6 (5.66) | 1 (0.31) | 14.333 | ＜0.001 |
|  | No | 100 (94.34) | 324 (99.69) |  |  |
| Leukoaraiosis | Yes | 59 (55.66) | 30 (9.23) | 105.158 | ＜0.001 |
|  | No | 47 (44.34) | 295 (90.77) |  |  |
| Higher risk of fall | Yes | 83 (78.30) | 98 (30.15) | 76.069 | ＜0.001 |
|  | No | 23 (21.70) | 227 (69.85) |  |  |
| Absence of collateral flow | Yes | 6 (5.66) | 0 (0.00) | 18.656 | ＜0.001 |
|  | No | 100 (94.34) | 325 (100.00) |  |  |
| Middle cerebral artery occlusion | Yes | 9 (8.49) | 4 (1.23) | 14.401 | ＜0.001 |
|  | No | 97 (91.51) | 321 (98.77) |  |  |
| Abnormal NIHSS | Yes | 76 (71.70) | 38 (11.69) | 147.941 | ＜0.001 |
|  | No | 30 (28.30) | 287 (88.31) |  |  |
| Abnormal GCS | Yes | 35 (33.02) | 76 (23.38) | 3.880 | 0.049 |
|  | No | 71 (66.98) | 249 (76.62) |  |  |
| Abnormal HAS-BLED | Yes | 76 (71.70) | 112 (34.46) | 45.065 | ＜0.001 |
|  | No | 30 (28.30) | 213 (65.54) |  |  |
| Anemia | Yes | 42 (39.62) | 113 (34.77) | 0.818 | 0.366 |
|  | No | 64 (60.38) | 212 (65.23) |  |  |
| Decreased platelet count | Yes | 7 (6.60) | 15 (4.62) | 0.652 | 0.419 |
|  | No | 99 (93.40) | 310 (95.38) |  |  |
| Abnormal liver function | Yes | 42 (39.62) | 92 (28.31) | 4.777 | 0.029 |
|  | No | 64 (60.38) | 233 (71.69) |  |  |
| Abnormal renal function | Yes | 26 (24.53) | 50 (15.38) | 4.601 | 0.032 |
|  | No | 80 (75.46) | 275 (84.62) |  |  |
| PT≥13.5 s | Yes | 82 (77.36) | 165 (50.77) | 23.097 | ＜0.001 |
|  | No | 24 (22.64) | 160 (49.23) |  |  |
| INR≥1.03 | Yes | 84 (79.25) | 172 (52.92) | 22.963 | ＜0.001 |
|  | No | 22 (20.75) | 153 (47.08) |  |  |
| APTT≥45.4 s | Yes | 40 (37.73) | 49 (15.08) | 25.045 | ＜0.001 |
|  | No | 66 (62.27) | 276 (84.92) |  |  |
| FIB≥4.20 g/L | Yes | 55 (51.89) | 88 (27.08) | 22.192 | ＜0.001 |
|  | No | 51 (48.11) | 237 (72.92) |  |  |
| TT≥18.3 s | Yes | 28 (26.42) | 54 (16.62) | 4.983 | 0.026 |
|  | No | 78 (73.58) | 271 (83.38) |  |  |
| D-dimer≥0.43 ug/mL | Yes | 84 (79.25) | 197 (60.62) | 13.538 | ＜0.001 |
|  | No | 22 (20.75) | 128 (39.38) |  |  |

BMI: body mass index; NSAIDs: non-steroidal anti-inflammatory drugs; MT: malignant tumor; VTE: venous thromboembolism; AF: atrial fibrillation; PVD: peripheral vascular diseases; TIA: transient ischemic attack; NIHSS: national institutes of health stroke scale; GCS: glasgow coma scale; PT: prothrombin time; INR: international normalized ratio; APTT: activated partial thromboplastin time; FIB: fibrinogen; TT: thrombin time.

**Supplementary Table 2. Results of multivariate analysis**

| **Variables** | **β** | **OR (95% CI)** | **P value** |
| --- | --- | --- | --- |
| Male | 2.218 | 9.185 (2.456-34.346) | 0.001 |
| Age ≥ 75 years old | -0.790 | 0.454 (0.099-2.081) | 0.309 |
| Dual antiplatelet therapy | -0.734 | 0.480 (0.029-7.843) | 0.606 |
| Beta blockers | -2.106 | 0.122 (0.024-0.605) | 0.010 |
| Diabetes | 0.669 | 1.953 (0.541-7.045) | 0.307 |
| Chronic liver disease or pancreatitis | -1.038 | 0.354 (0.042-2.960) | 0.338 |
| Esophageal varices | -17.746 | <0.001 (<0.001-/) | 0.999 |
| History of major bleeding | 0.076 | 1.079 (0.124-9.371) | 0.945 |
| VTE | -0.573 | 0.564 (0.105-3.018) | 0.503 |
| Cardiogenic embolism | -1.331 | 0.264 (0.009-7.784) | 0.441 |
| Small vascular diseases | -0.725 | 0.484 (0.041-5.746) | 0.566 |
| Lacunar infarction | 1.563 | 4.775 (0.379-60.104) | 0.226 |
| Cerebral microbleeds | 1.158 | 3.185 (<0.001-/) | 1.000 |
| Arteriosclerosis | 1.492 | 4.448 (0.721-27.450) | 0.108 |
| Cardiovascular diseases | 0.018 | 2.769 (0.207-37.049) | 0.442 |
| AF | 0.060 | 1.062 (0.289-3.907) | 0.928 |
| Hypertension | -0.706 | 0.494 (0.125-1.955) | 0.315 |
| PVD | -0.980 | 0.375 (0.093-1.518) | 0.169 |
| Cerebrovascular diseases | -15.825 | <0.001 (<0.001-/) | 0.999 |
| Stroke or TIA | 16.854 | 20873555.12 (<0.001-/) | 0.999 |
| Intracranial aneurysm | 20.464 | 771615921.00 (<0.001-/) | 0.999 |
| Leukoaraiosis | 2.004 | 7.416 (1.706-32.230) | 0.008 |
| Higher risk of fall | 2.683 | 14.622 (3.520-60.746) | <0.001 |
| Absence of collateral flow | 18.309 | 89424678.29 (<0.001-/) | 0.999 |
| Middle cerebral artery occlusion | -0.893 | 0.409 (0.045-3.701) | 0.427 |
| Abnormal NIHSS | 23.900 | 2.397ⅹ10^10^ (<0.001-/) | 0.995 |
| Abnormal GCS | -20.475 | <0.001 (<0.001-/) | 0.995 |
| Abnormal HAS-BLED | 0.766 | 2.151 (0.531-8.719) | 0.283 |
| Abnormal liver function | 1.426 | 4.160 (0.589-29.383) | 0.153 |
| Abnormal renal function | -0.325 | 0.723 (0.168-3.113) | 0.663 |
| PT≥13.5 s | 1.175 | 3.239 (0.395-26.523) | 0.273 |
| INR≥1.03 | 0.157 | 1.170 (0.127-10.762) | 0.890 |
| APTT≥45.4 s | 1.219 | 3.384 (1.038-11.035) | 0.043 |
| FIB≥4.2 g/L | 1.819 | 6.165 (1.820-20.876) | 0.003 |
| TT≥18.3 s | 0.018 | 1.018 (0.252- 4.108) | 0.980 |
| D-dimer≥0.43 ug/mL | 0.564 | 1.757 (0.424-7.281) | 0.437 |

VTE: venous thromboembolism; AF: atrial fibrillation; PVD: peripheral vascular diseases; TIA: transient ischemic attack; NIHSS: national institutes of health stroke scale; GCS: glasgow coma scale; PT: prothrombin time; INR: international normalized ratio; APTT: activated partial thromboplastin time; FIB: fibrinogen; TT: thrombin time.
